# Emotion Regulation, Cognition, and Well-Being Across the Adult Lifespan

**DOI:** 10.1093/geroni/igaf122.047

**Published:** 2025-12-31

**Authors:** Hannah Wolfe, Derek Isaacowitz

## Abstract

This symposium explores the multifaceted nature of emotion regulation (ER) and its relationship with cognition and well-being across the adult lifespan. Wolfe and Isaacowitz investigated the cognitive demand and outcomes of ER tactics in younger and older adults. They found that positive-approaching was self-reported as the most demanding tactic and was linked to the highest memory accuracy; yet, there were no differences in pupil dilation across tactics. Growney & English further examined cognitive demands of ER strategies in younger adults, cognitively normal (CN) older adults, and older adults with mild cognitive impairment (MCI). They found that positive-emotion-enhancing strategies were perceived as less demanding and that younger and CN older adults, but not older adults with MCI, used high-demand strategies less frequently. Lai et al. explored whether ER abilities assessed in the lab align with success in daily life. Their results indicated minimal associations between lab and daily-life ER success across younger adults, CN older adults, and older adults with MCI, suggesting that lab-based assessments might capture different facets of ER ability. They also found age-related advantages in daily ER success, including among older adults with MCI. Finally, Britton et al. broadened this perspective by analyzing links between psychological well-being and cognitive function in older adulthood. They found that multiple components of well-being, including sense of purpose and self-acceptance, were significantly positively associated with both concurrent and future cognitive function, suggesting that psychological well-being may contribute to the cognitive resources involved in effective ER. Derek Isaacowitz will serve as a discussant.

